# Digital transformation and middle managers’ leadership skills and behavior: a group concept mapping approach

**DOI:** 10.3389/fpsyg.2023.1147002

**Published:** 2023-09-04

**Authors:** Maartje Henderikx, Jol Stoffers

**Affiliations:** ^1^Research Centre for Employability, Zuyd University of Applied Sciences, Sittard, Netherlands; ^2^Faculty of Educational Sciences, Open University of the Netherlands, Heerlen, Netherlands; ^3^Faculty of Management, Open University of the Netherlands, Heerlen, Netherlands; ^4^Research Centre for Education and the Labour Market (ROA), Maastricht University, Maastricht, Netherlands

**Keywords:** digital transformation, middle managers, leadership skills, leadership behavior, group concept mapping

## Abstract

This study, with the aim to test theory in practice, used group concept mapping to develop a comprehensive conceptualization of middle managers’ leadership behaviors concerning digital transformation as a form of radical change. Participants were professionals in the largest public organization in the Netherlands (a police organization) who were dealing with digital transformation in their own practice and who enrolled in an education program on leadership and intelligence. Based on 94 unique statements, the participant-driven results revealed six thematically coherent clusters representing leadership skills and behaviors regarding improvement and results, digital technologies, cooperation, the self, change and ambivalence, and others. The stress value of 0.2234 indicated a good fit. Further analysis showed that clusters containing soft skills and people-oriented behaviors were considered the most important. These results can serve as input to support leadership development programs for middle managers to develop themselves into people-oriented, empowering leaders who can adapt their leadership approaches to fit and support change in general and technology-driven change in particular. Ultimately this will benefit their and their employees’ overall well-being at work. This study is the first to investigate middle managers’ leadership skills and behaviors in a large public organization that is entirely participant-driven.

## 1. Introduction

Affected by the increasing emergence and influence of technological innovation, digital transformation is a top priority for contemporary organizations as technology continues to shape personal and professional lives (Roblek et al., 2021; Volberda et al., 2021), driving technology-driven disruptive change (Verhoef et al., 2021; Henderikx and Stoffers, 2022). People generally classify digital transformation as a radical change, like globalization and deregulation, since it is still a relatively new and rapidly evolving phenomenon whose full impact is not yet understood (Westerman et al., 2014; Christensen et al., 2015). Due to its imminent rise, many studies focus on digital transformation and leadership, exploring emergence-related challenges of strategic leadership (Vial, 2019; Kurzhals et al., 2020; Tetik, 2020). Since digital transformation is common in many organizations, it is increasingly evident that such leadership challenges are relevant across all management levels (Petry, 2018; Nadkarni and Prügl, 2021). This includes the middle management level, as middle managers are essential to leading and supporting organizational change (Vial, 2019; Henderikx and Stoffers, 2022). Middle managers are charged with converting organizational strategies into daily practices, requiring in-depth knowledge of the organization and the connections that span its levels (Stoker, 2006). Once digitization deploys, they must facilitate its process while continuing to lead and manage a new digital organization (Klein, 2020). However, middle management has thus far received little attention (Nadkarni and Prügl, 2021) when it comes to conceptualizing leadership to oversee such transformations (Kaivo-Oja et al., 2017; Gfrerer et al., 2021; Wu et al., 2021).

Despite the importance discussed above, the influence of digital transformation on middle management leadership level remains underexplored (Nadkarni and Prügl, 2021; Henderikx and Stoffers, 2022). To fill the gap in existing literature, Henderikx and Stoffers (2022) conducted a literature study into digital transformation and middle management leadership. They found that particularly soft skills and understanding the power of digital technology were becoming increasingly important. The present study builds on this literature study by testing theory in practice with the actual target group, the middle manager. The focus is determining which leadership skills or behaviors middle managers deem essential in light of the ongoing digitalization.

Generally, this is a difficult task due to the shortcomings of using either traditional quantitative or qualitative methods. However, group concept mapping (GCM) as a participant-driven mixed-methods approach, represents a potentially valuable concept. The participatory approach maximizes numerous knowledge sources (Trochim, 1989), by including the knowledge and ideas of stakeholders on multifaceted issues in a structured process. In addition, the approach fosters the effective realization of future interventions by engaging these stakeholders at an early stage. It is particularly effective when applied to complex, comprehensive topics, such as understanding social innovation leadership in universities (Milley and Szijarto, 2020) or digital transformation in SMEs from an ecosystemic viewpoint (Pelletier and Cloutier, 2019), because it enables a detailed overview of various components. In addition, based on a pooled analysis of 69 studies by Rosas and Kane (2012), GCM provides robust internal representational validity and effective sorting and ranking reliability estimates.

This article reports on a GCM study that offers a comprehensive conceptualization of relevant leadership behaviors and skills according to middle managers who were (and still are) dealing with digital transformation in their own practice at the time of the data collection. This study’s results deepen our understanding of this particular topic and supplement extant leadership research on radical change in general. First, we discuss the theoretical background, after which the research approach is outlined, and results from initial analyses are reported. The subsequent discussion section examines these results, highlighting their consequences. Lastly, limitations and potential topics for future research on to build are discussed.

## 2. Theoretical background

### 2.1. Middle management

From various studies across different industries, it is widely agreed that middle managers are important for the success of an organization (Engle et al., 2017; DeFilippis et al., 2022). They play a crucial role in instigating change across the organization by influencing the mentalities and reasoning of their employees, as highlighted by Reynders et al. (2020) and Tsuda and Sato (2020). Their function strongly influences the implementation of entire strategic directions, as Kieran et al. (2020) emphasized. The benefits of a clearer understanding of how middle managers affect organizational transformation have been increasingly highlighted in recent years (Reynders et al., 2020; Henderikx and Stoffers, 2022; Van Doorn et al., 2022). Huy (2001, p. 73) characterizes middle managers as one level above line managers and two below the CEO. As opposed to senior managers’ goal- and policy-setting functions, middle managers ensure the strategic execution of operations (Reynders et al., 2020), including reacting to the rapid changes and complexities, digital ecosystems bring. To achieve proactive digital transformations, it is crucial to entrust important tasks to middle managers who understand international markets and directly engage with stakeholders like customers in the digital environment. This ensures effective collaboration throughout the process (Alieva and Powell, 2022). During such transformations, middle managers must cope with innovative cultures, internal knowledge absorption, dynamic external environments, and rapidly changing internal organizational identities (Volberda et al., 2021; Alieva and Powell, 2022). The purpose of middle managers requires rethinking that aligns with the evolving digital ecosystems (Volberda et al., 2021), whereby they support ongoing digitalization and ultimately manage and lead emerging digital organizations (Klein, 2020).

### 2.2. Leadership skills and behaviors and digital transformation

Due to the increased complexity that digital ecosystems bring, traditional leadership approaches do not suffice; leading, guiding, and managing during digital transformations require a reassessment of leadership skills, behaviors, and new understandings of leadership (Mirhosseini et al., 2020). Digitalization has indeed triggered new leadership paradigms like e-leadership and digital leadership, but these approaches generally focus on the digital workforce and the use of digital assets (Torre and Sarti, 2020; Araujo et al., 2021). Recent studies identify the importance of a shift from top-down leadership to people-oriented leadership approaches (Ready et al., 2020; Henderikx and Stoffers, 2022). Studies that assess both leadership and digital transformations suggest a growing need for soft skills and behaviors (e.g., Klus and Müller, 2020; Ready et al., 2020; Henderikx and Stoffers, 2022), in combination with digital intelligence (Cortellazzo et al., 2019; Boughzala et al., 2020).

#### 2.2.1. Soft skills

Soft skills are “personal attributes that enable someone to interact effectively and harmoniously with other people” (Oxford University Press, 2022), representing behaviors and attitudes that enable interactions with others relationally (Lista et al., 2022). They include emotions, values, and perspectives, which are challenging to share with and transfer to others (Badurdeen et al., 2010; Bauer et al., 2018). Emotions related to soft (leadership) skills were found to increase employee well-being (Inceoglu et al., 2018; Dong and Yan, 2022; Caniëls, 2023). Soft leadership behaviors that appear increasingly important are empathy, flexibility, adaptability, integrity, vulnerability, tolerance, and patience (Jakubik and Berazhny, 2017; Klus and Müller, 2020; Ready et al., 2020; Henderikx and Stoffers, 2022).

#### 2.2.2. Digital intelligence

The acquisition of digital intelligence involves the ability to read, modify, and interpret digital data, as well as to derive meaning and make informed decisions based on that data (Cortellazzo et al., 2019; Boughzala et al., 2020; El Sawy et al., 2020). To succeed in their role both now and in the future, middle managers should possess digital intelligence. Employees with digital intelligence are not just skilled in using technology, but also possess a deep understanding of how it can improve operational efficiency and outcomes (Boughzala et al., 2020). By continuously updating their knowledge and skills related to digital technology, they are able to drive innovation in their organizations (Cortellazzo et al., 2019).

#### 2.2.3. Additional skills and behaviors

Moving further into the digital age, it’s becoming increasingly clear that organizations need to be able to keep up with the rapid pace of technological change. To do so, employees must be prepared to adapt to new situations, be comfortable with ambiguity, and be willing to experiment and take risks which are typically skills and behaviors that belong to dealing with change (El Sawy et al., 2020). A recent literature study into digital transformation and leadership by Henderikx and Stoffers (2022) highlights that middle managers in particular must develop themselves as people-oriented, technically-minded, empowering leaders who are able to adjust their leadership approaches to fit the needs of the situation at hand.

## 3. Methods

### 3.1. Group concept mapping

**Figure 1:**
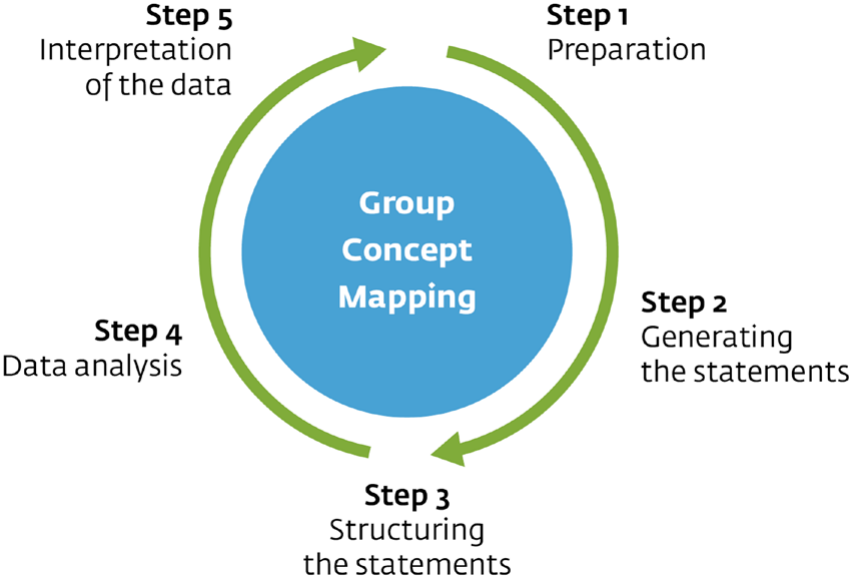
Schematic overview of Group Concept Mapping process. Based on Hagell et al. (2016).

GCM is a mixed-methods approach that allows gaining insights into a group’s understanding of multifaceted phenomena and discovering new meanings (Kane and Trochim, 2007). Rosas and Kane (2012), van Bon-Martens et al. (2014), Rosas and Ridings (2017), and Trochim and McLinden (2017) extensively described the method. Therefore, we will only provide a summary here. According to Trochim (1989), using participant-driven data, GCM creates visualizations representing a target group’s ideas and opinions. The process consists of five steps—preparation, generating statements, structuring statements, data analysis, and data interpretation (Figure 1).

Step 1 involves selecting participants and choosing a focus for the study. The researcher then formulates a focus prompt to guide the brainstorming phase and criteria for rating the statements. The rating criteria depend on the purpose of the study. During Step 2, the brainstorming phase, participants generate statements by completing the formulated focus prompt multiple times. The exercise ideally results in a varied, saturated set of statements that covers the focus of the study. A researcher typically edits the statements, checking for duplicates, split statements containing more than one thought and remove statements irrelevant to the study’s focus. An optimal set contains 80 to 100 statements (Trochim and McLinden, 2017).

During Step 3, the statements are sorted and rated. The participants sort the statements into piles with similar meaning or relevance and then label these piles. Restrictions on sorting include (1) a statement cannot be sorted into multiple piles, (2) more than one pile must be made, and (3) each pile must contain more than one statement. Jackson and Trochim (2002) recommend that a minimum of 10 sorters is needed, and Rosas and Kane (2012) suggest that 20 to 30 sorters is optimal. However, regardless of the number of sorters, a stress value—a fit indicator calculated using GCM software—between 0.205 and 0.365 indicates a good fit of the group concept mapping representation (Rosas and Kane, 2012). The rating of the statements occurs after sorting the statements generally using a Likert-type scale. Based on the rating criteria determined during Step 1, participants rate the statements in relation to each other.

During Step 4, data generated by the participants during Step 3 are analyzed with GCM software using multidimensional scaling (MDS) and hierarchical cluster analysis, resulting in 2-dimensional point and cluster maps. Points (i.e., statements) that are proximate on the map are sorted together more frequently, and vice versa (Trochim, 1989). Points grouped together represent clusters, and an iterative process, conducted by a researcher, determines the optimal amount of clusters with meaningful content. Lastly, during this step, average ratings of the statements from participants are combined with the generated point map and the cluster map, which results in layered versions of these maps. The layers represent participants’ average ratings of the statements in other words, how important they feel the cluster is. Overall, analysis in step 4 results in four interpretable visualizations—the point map, the cluster map, the point rating map, and the cluster rating map. Step 5 involves the interpretation of the data analyzed during Step 4.

### 3.2. Participants

Participants were professionals at the largest public organization in the Netherlands (a police organization) who were and still are dealing with digital transformation. They enrolled in an education program on leadership and intelligence to better understand leadership in the light of (digital) change. The program ran from March to December 2021. All 40 participants (11 female, 29 male, middle and higher management) were invited to contribute to the brainstorming task of the study, of whom 25 (6 female, 19 male) completed the task. Only middle managers (15 participants, 3 female, 12 male) were included during the sorting and rating task since they are the focus of this study.

### 3.3. Procedure

Participants were invited to participate in this study through the leadership course. Brainstorming, sorting, and rating tasks were completed online and were integrated into the study’s structure separately. The participants received an invitation which explained the data collection procedure. The invitation also emphasized that participation was voluntary and not conditional to completing the course. Before brainstorming using the online tool, participants were again informed that participation was voluntary and asked to give their informed consent if they decided to participate.

Within 2 weeks, the participants could generate statements based on a focus prompt—“Important leadership skills and behaviors in view of the ongoing digitalization of your work are or will be….” They also responded to questions about their gender and management level. After 2 weeks, the participants generated 87 statements. The researchers then edited the statements and removed redundant ones (Trochim, 1989), after which 61 remained. To ensure a varied and saturated set of statements, the researchers added 23 statements from literature on leadership behaviors and skills and digital transformation (see Appendix A), resulting in 94 statements available for sorting and rating.

After the brainstorming, the participants had 4 weeks to complete the sorting and rating tasks. The participants first sorted the statements into piles of similar meaning or relevance. Then they rated the statements according to their perceived importance compared to the other statements using a 5-point, Likert-type scale ranging from very unimportant to very important (5). Participants who completed less than 75% of the sorting and rating were excluded from the analysis (Schophuizen et al., 2018). After the participants completed these tasks, the researchers examined and selected the optimal number of cluster solutions supported by the online GCM tool. The aim was to achieve a cluster combination that best represented the data in combination with internal thematic cluster coherence. This process resulted in six interpretable clusters that were subsequently labeled with names representing their thematic content.

## 4. Results

### 4.1. Point map

**Figure 2:**
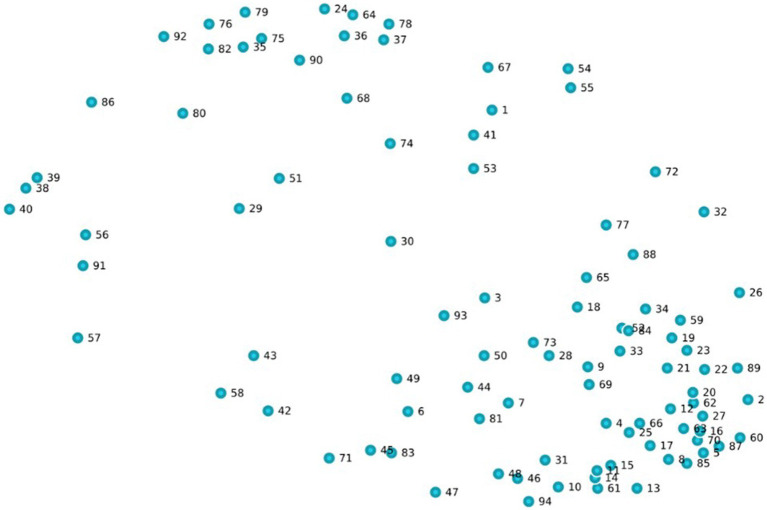
Point map of the 94 statements.

Combining the final set of generated statements with additional statements from theory resulted in 94 unique statements. Figure 2 shows the point map of this set. Each statement can be identified by number (see Appendix A). The point map illustrates the relational structure of the statements based on participants’ input (Trochim and McLinden, 2017).

The GCM tool calculated the distance between points based on the bridging values of each point. A bridging value indicates the proximity of statements to each other and can range from zero to 1. Statements with lower bridging values have been sorted together in piles more often by participants and are therefore grouped together on the map, indicating a similar meaning or theme (Trochim and McLinden, 2017). The calculated stress value determined how well the visualization of the point map fits the data. Generally, stress values within the range of 0.205 to 0.365 are acceptable (Rosas and Kane, 2012). The average stress value was 0.2234 after 14 iterations, which suggests that the map is a good representation of the sorting data.

### 4.2. Cluster map

**Figure 3:**
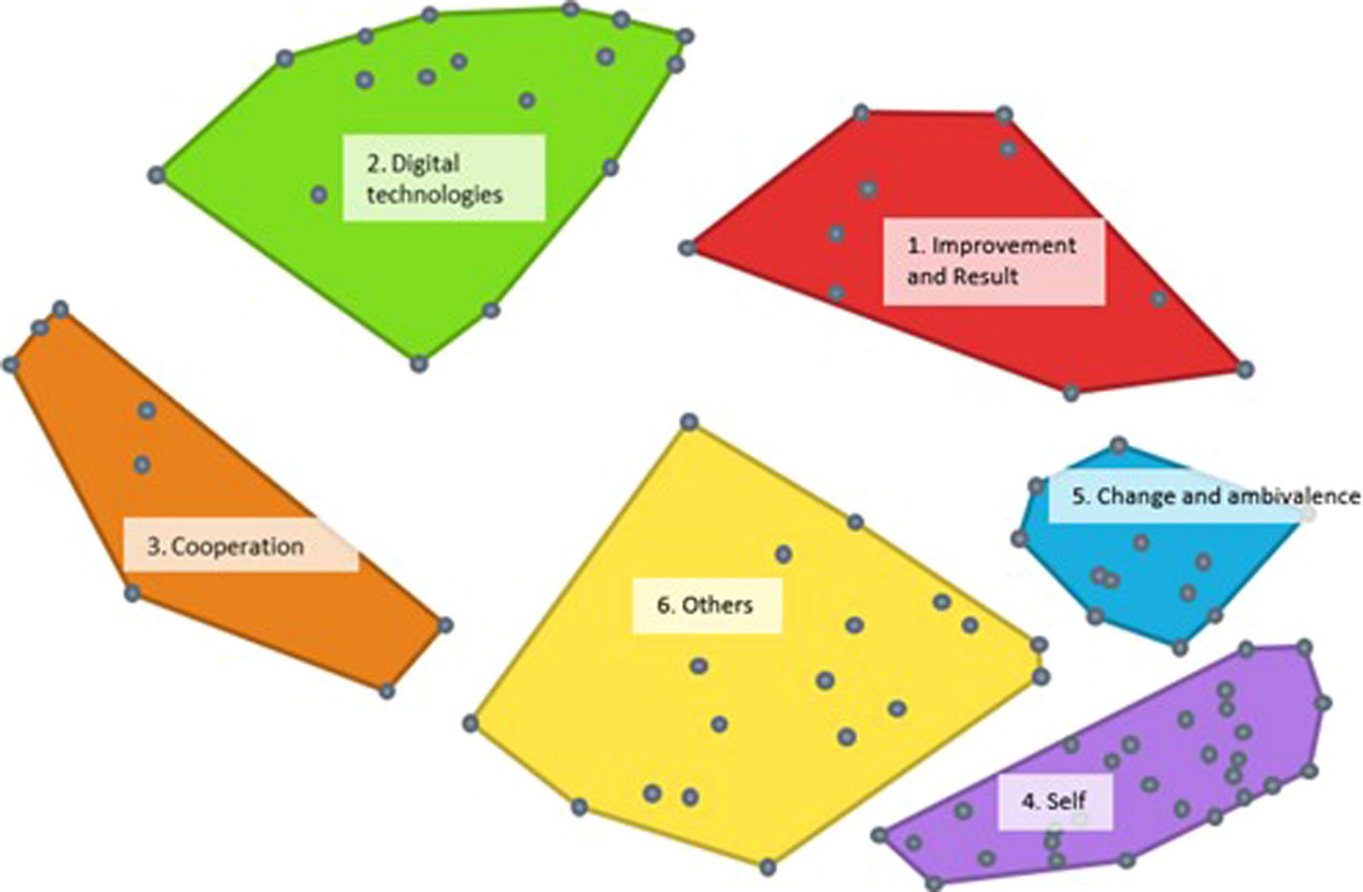
Cluster map of the 94 statements. Dots may represent multiple statements.

The point map in Figure 2 is the basis for the hierarchical cluster analysis. After considering multiple options, a six-cluster solution was selected as optimal. After the researchers investigated the cluster content, five statements were reassigned to neighboring clusters based on better conceptual fit (see Appendix A). This solution sorted the data best into interpretable and distinct clusters labeled according to leadership skills and behaviors, including improvement and results, digital technologies, cooperation, change and ambivalence, self, and others (Figure 3).

Each statement was assigned a bridging value, and the values were aggregated and averaged per cluster. When participants often sorted statements together, the mean bridging value of a cluster was low. Table 1 presents an overview of the number of statements per cluster and the average bridging value and range per cluster.

**Table 1:** Overview of cluster information.

No.	Cluster	Number of statements	Mean bridging value	Bridging value range
1	Improvement and results	10	0.54	0.39–0.65
2	Digital technologies	17	0.49	0.29–0.72
3	Cooperation	8	0.86	0.62–1.00
4	Self	29	0.07	0.02–0.22
5	Change and ambivalence	12	0.20	0.07–0.36
6	Others	18	0.28	0.06–0.62

The cluster with the greatest thematic coherence was Cluster 4, which contains leadership skills and behaviors regarding the self. The cluster with the lowest thematic coherence is Cluster 3, which contains skills and behaviors regarding cooperation.

Cluster 1 contains 10 statements related to leadership skills and behaviors regarding improvement and results which are important concepts with regard to organizational success. Improvement refers to the process of making something better, results refers to the outcomes of that process. Bridging values range from 0.39 to 0.65 (M = 0.54), suggesting the cluster is thematically coherent. The cluster contained one statement that was added from theory (see Appendix A), and one statement, “lead by results,” was moved from the neighboring Cluster 5 to this cluster for better conceptual fit. In addition to general behaviors and skills, such as “lead by results” and “focus on performance,” this cluster contains behaviors and skills that are typical in the context of digitalization, such as “being able to support online teams,” “stimulate remote working,” “taking opportunities to improve work,” and “evaluate digital work activities.”

Cluster 2 contains 17 statements on leadership skills and behaviors regarding digital technologies. This refers to knowledge about and the ability to effectively use digital technologies to lead and manage. The bridging values ranging from 0.29 to 0.72 (M = 0.49). Like Cluster 1, the bridging values indicate reasonable thematic coherence and sorter consensus. The cluster contains statements related to working with digital technologies (e.g., “being able to structure information using digital technologies”), awareness of digital technologies’ positive and negative issues (e.g., “monitoring online privacy and security”), and recognizing digital technologies’ potential and value (e.g., “being aware of the possibilities of informatics and robotics”).

Cluster 3 was the smallest, containing eight statements on leadership skills and behaviors regarding cooperation which refers to working together with others to achieve a common goal. Bridging values range from 0.62 to 1.00 (M = 0.86), suggesting a diverse cluster; participants sorted these statements inconsistently. Example statements include “encourage international cooperation” and “invest in external cooperation regarding surveillance in digital environments.” Two statements—“stimulate internal cooperation” and “foster interpersonal communication”—were moved from neighboring Cluster 6 to this cluster for improved conceptual fit.

Cluster 4, the largest, contains 29 statements on leadership skills and behaviors regarding the self. These statements are related to an individual leader’s personal development and growth which positively affect leadership. Thirteen statements were added from theory (see Appendix A). Bridging values range from 0.02 to 0.22 (M = 0.07), suggesting a very thematically coherent cluster; participants agreed greatly regarding which leadership skills and behaviors a middle manager should possess during ongoing digitalization. Examples include “authentic,” “inspiring,” “dedicated,” “good sense of ethics,” and “open to feedback.”

Cluster 5 contains 12 statements on leadership skills and behaviors regarding change and ambivalence. These are typically skills and behaviors that support effectively managing change and the uncertainty that often accompanies it and can help overcome resistance to change, build support for change, and create a positive environment for change. Four statements from theory were included (see Appendix A). Bridging values range from 0.07 to 0.36 (M = 0.20), which suggests a very coherent cluster. The skills and behaviors mentioned in the statements related to changing circumstances and innovation include “not afraid to fail,” “recognize opportunities,” “able to cope with resistance,” and “able to make quick decisions.”

Cluster 6, the second largest, includes 18 statements on leadership skills and behavior regarding others These statements center around a leader’s ability to effectively interact with and influence others and build strong relationships, motivate others, and create a positive work environment. Four were added from theory (see Appendix A). Bridging values range from 0.06 to 0.62 (M = 0.28), suggesting a moderately coherent cluster. Participants grouped these skills and behaviors because they involved dealing with others; they are about supporting employees at work regarding professional and personal development, including “empowering people” and “enthusing employees,” or about coping with others, including “communicating effectively” and “being sensitive to feelings.”

### 4.3. Point rating map

**Figure 4:**
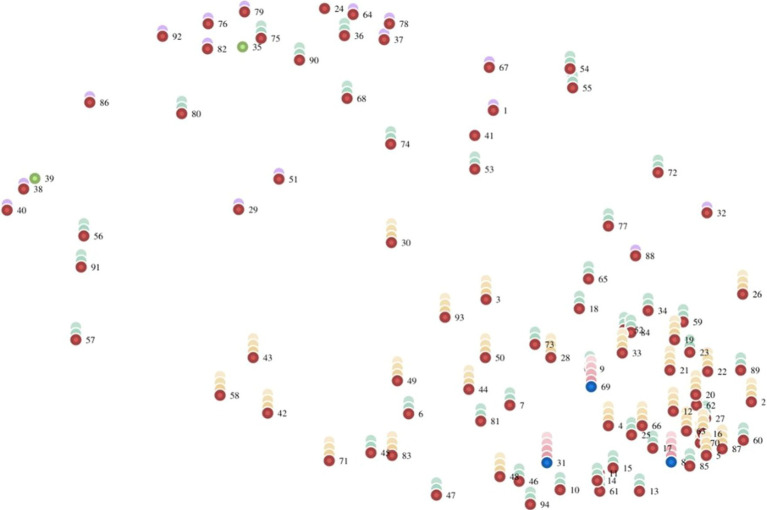
Point rating map showing the average importance of the statements. Green dots represent the lowest-rated statements (less than 3.40; #11 [not visible, behind #14], 35, 39). Blue dots represent the highest-rated statements (greater than 4.53; #8, 31, 69).

The point rating map is based on the point map described in 4.1 but includes the average ratings per statement. During rating, participants rated each statement based on perceived importance in relation to the other statements using a Likert-type scale. Colored stacks in Figure 4 represent the ratings; the higher the stack, the more important the participants perceived the statement.

Table 2 reports the three skills and behaviors participants considered least and most important. The least and most important skills appear in Cluster 4, which relates to the self.

**Table 2:** Lowest- and highest-rated statements regarding perceived importance.

No.	Lowest-rated statement	Highest-rated statement	M	SD	Cluster
11	Humbleness		3.07	0.85	Self
35	Knowledge of digital tools, assets, software, and hardware		3.27	0.85	Digital technologies
39	Investing in external cooperation regarding enforcement in digital environments		3.34	0.79	Cooperation
8		Integrity	4.87	0.34	Self
31		Trust	4.60	0.49	Self
69		Empower people	4.53	0.50	Others

### 4.4. Cluster rating map

**Figure 5:**
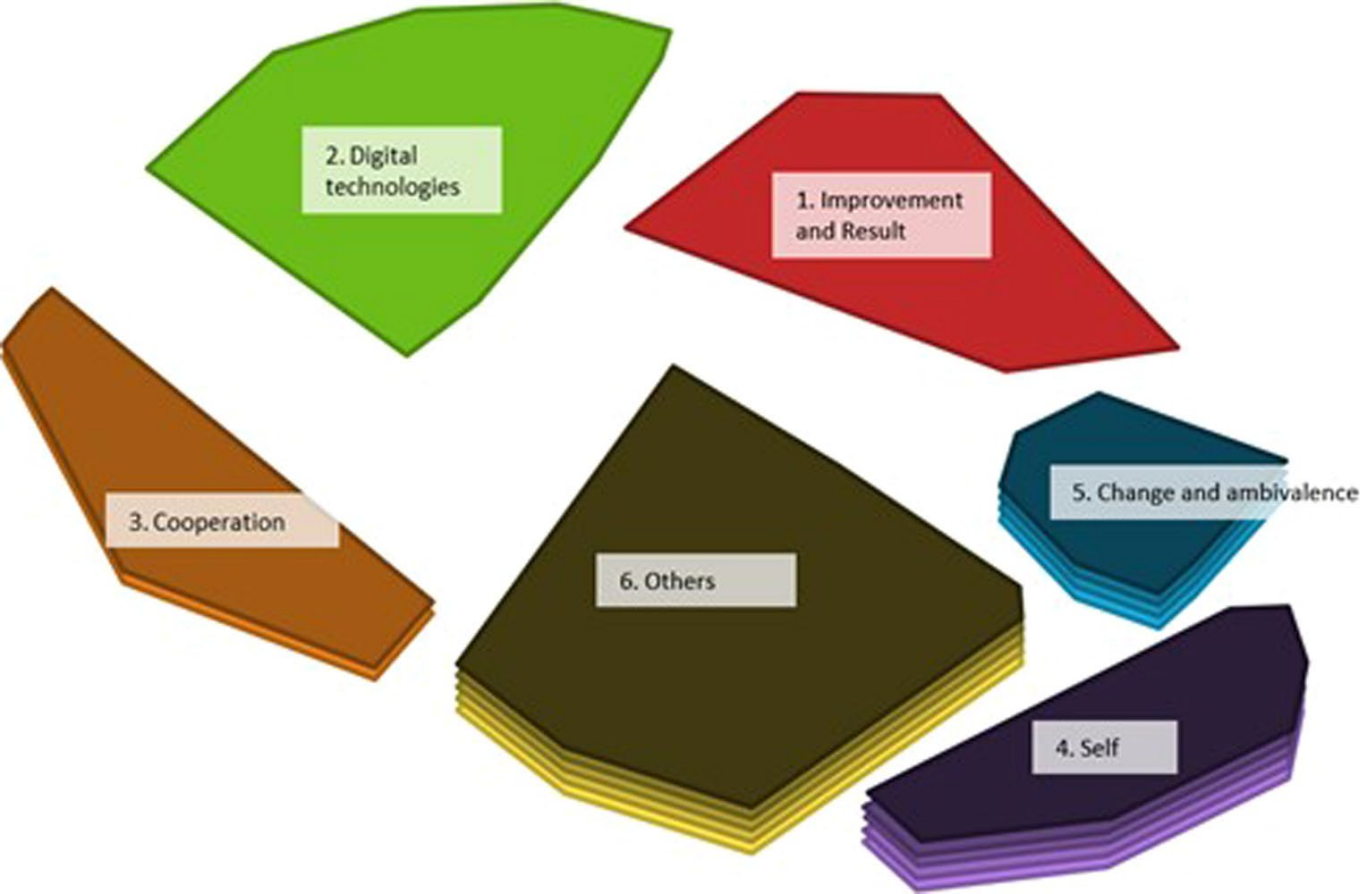
Cluster rating map showing the average importance of the clusters.

The cluster rating map is also based on participants’ ratings of the statements but includes the average perceived importance ratings across all statements per cluster (Figure 5). The number of layers in a cluster indicates its perceived importance; the more layers, the more important participants perceived it.

Based on the layers, Clusters 4 and 6 were considered the most important, and Clusters 1 and 2 the least. This finding is supported by the average perceived importance ratings per cluster, reported in Table 3.

**Table 3:** Average ratings per cluster regarding importance.

No.	Cluster	Mean	SD
1	Improvement and results	3.77	0.26
2	Digital technologies	3.69	0.21
3	Cooperation	3.88	0.34
4	Self	4.14	0.32
5	Change and ambivalence	4.08	0.20
6	Others	4.24	0.18

Cluster 2, which contains skills and behaviors regarding digital technologies, has the lowest mean score, and Cluster 6, which relates to others, has the highest mean score.

## 5. Discussion

### 5.1. Reflection on the outcomes

Using group concept mapping, this study identified which leadership skills and behaviors are important to middle managers in the context of ongoing digitalization. Multiple studies suggest that middle managers in larger public organizations must focus on employees by supporting them through (digital) change (e.g., Petry, 2018; Henderikx and Stoffers, 2022). Since most leadership research does not assess middle managers, this study adds to the literature by assessing conceptions of middle managers’ leadership skills and behaviors in relation to digital transformation to deepen understanding of this topic and supplement extant leadership studies. In response to this gap in the literature, the objective was to identify which leadership skills and behaviors are considered necessary during ongoing digitalization, according to middle managers, the management layer that is essential to leading and supporting organizational change (Vial, 2019; Reynders et al., 2020).

The participant-driven results revealed six very to reasonably thematically coherent clusters. These clusters represent a broad array of leadership skills and behaviors primarily based on the statements generated and sorted by the participants of the study. The clusters cover leadership skills and behaviors regarding improvement and results, digital technologies, cooperation, self, change and ambivalence, and others. Subsequent ratings demonstrated that participants considered integrity, trust, and empowering people as the most important skills and behaviors. Humbleness, having knowledge of digital tools/assets/software/hardware, and investing in external cooperation regarding enforcement in digital environments were considered the least important. No skill or behavior was rated unimportant (less than 3 on a 1 to 5 point Likert scale). Connecting this finding to cluster levels, the middle managers rated the clusters of self and others as most important and digital technologies and improvement and results as least important.

It is surprising that the digital technologies cluster was rated least important, considering that middle managers are commonly tasked with facilitating digitalization and leading and managing a digitally transformed organization, as noted by Klein (2020) and Reynders et al. (2020). One explanation is that participants were middle managers who worked in a large (public) organization, in which, due to its size, middle managers are typically concerned with overseeing day-to-day operations. Consequently, they often delegate duties of specific expertise to specialized departments equipped with the necessary skills to implement new digital technologies in business processes (e.g., technical; Vaccaro et al., 2012). It is, therefore, less vital for middle managers working in large organizations to acquire particularly hard skills regarding digital technologies as it enables them to concentrate on their primary responsibilities while ensuring the organization keeps pace with digital advancements. This may be why they consider such skills less important compared to other skills and behaviors. Nonetheless, middle managers still play a vital role in digital transformation, bridging technical experts and the operational staff (Giauque, 2015). They are responsible for translating the implications of digital changes into actionable steps and guiding their teams through these transitions.

Clusters that contained soft skills, such as interactions that enable people-oriented skills and behaviors, were considered the most important. These results align with those from Henderikx and Stoffers (2022), who argue the need for soft skills as they are inherently human and complex to replicate through technological advancements and automation. Technology does not (yet) encompass human-like emotions, values or perspectives, which are at the heart of soft skills (Badurdeen et al., 2010; Bauer et al., 2018). For example, increasingly working with digital tools and working remotely has made soft skills such as empathy and clear communication vital in fostering effective collaboration (e.g., Cortellazzo et al., 2019; Klus and Müller, 2020). Also, digital transformation often involves frequent changes, the implementation of new technologies, and shifting priorities. Soft skills like adaptability and flexibility make it possible to embrace these changes, quickly learn new tools and processes, and navigate through uncertainty (e.g., Jakubik and Berazhny, 2017). Furthermore, as organizations undergo digital transformation, strong, soft skills to inspire and motivate employees and teams are essential (e.g., Ready et al., 2020). Overall, soft people-oriented skills are essential for building effective relationships, adapting to change, fostering innovation, and leading successful digital initiatives and teams (e.g., Lista et al., 2022). Therefore, the importance of nurturing and developing these soft skills alongside technological advancements should be recognized. Overall, we can conclude that what the middle managers in this study regard as relevant leadership behavior and skills is consistent with the findings of the literature study it is built on as well as consistent with other studies in this direction, for instance, from Jakubik and Berazhny (2017), Klus and Müller (2020), and Ready et al. (2020).

Enabling middle managers to develop themselves as people-oriented, technically minded, empowering leaders who can adapt their leadership to fit with changing (digital) circumstances is therefore essential. Especially as leadership behavior, precisely behavior related to soft skills positively impacts employee well-being (e.g., Inceoglu et al., 2018; Dong and Yan, 2022), even more so with the current increase of remote working (Caniëls, 2023). The development of soft skills in middle managers can be seamlessly incorporated into daily operations through the novel use of simulations related to social issues alongside habitual practice and reinforcement in real-life scenarios. These simulations can concurrently cultivate both soft skills—emphasizing societal and human aspects—and hard skills—focusing on technical and conceptual facets (Poisson-de Haro and Turgut, 2012). Managers should apply active listening, empathy, and effective communication during daily interactions, with simulations offering a safe environment for practice. A culture encouraging feedback and reflection can boost continuous learning (Tripathy, 2020). The necessity for such comprehensive skills development is paramount given middle managers’ crucial role, as it enhances team cohesion, improves decision-making, and ultimately propels organizational success.

### 5.2. Practical implications

Middle managers serve as a vital bridge between strategic and operational levels, managing the translation of strategies into actions and addressing immediate operational concerns (Ekaterini, 2011). This makes them indispensable to the smooth functioning of public organizations. Moreover, competition among experienced managers, uncertain economies, failures in public organizations, and rapidly changing demographics and digitalization requires developing talented, in-house employees to become effective leaders (Gusain, 2017; Holt et al., 2018). Creating effective leadership development strategies, especially in large public organizations, requires an in-depth understanding of the internal and external challenges that organizations experience (Moldoveanu and Narayandas, 2019). We advocate an approach in which top managers’ roles and attitudes align with enhancing structures and strategies essential to improving middle managers’ leadership development. This is especially true since management in large public organizations is hierarchical and formal. Also, identifying leadership requirements and allocating necessary (financial) resources for development is paramount. HR departments thus must facilitate and monitor the entire process, preferably data-driven.

Generally, an effective leadership development program includes a curriculum that focuses on teamwork, collaboration, and communication skills and emphasizes each potential talent’s unique personality Ideally, it should run for approximately 2 years, during which classroom and online learning should be alternated, in addition to daily practical experiences (Holt et al., 2018). A (scientifically substantiated) individual quick-scan leadership assessment tool, preferably designed as a “self-other” assessment (as a form of multi-source evaluation; Alexandru and Diana, 2015), could support such programs. This assessment tool can reinforce data-driven leadership development cycles in the organization by determining the starting point as well as monitoring individual (leadership) development progression (Vukotich, 2010). More specifically, in the context of this study, a leadership development program requires an understanding of the dynamic digital landscape and prioritization of soft skills. The program should focus on training middle managers in digital tools and technologies while enhancing their interpersonal skills for better team management. Interactive modules and experiential learning can support nurturing behaviors favorable to leading in a digital age. Using the increasing availability of online courses, social platforms, and learning tools. Both traditional education providers and new startups offer these resources. They are helping to better meet the needs of organizations, and individual learners. Moldoveanu and Narayandas (2019) provide a significant analysis of the diverse institutions operating within the realm of executive education. They posit that a new wave of competitors is surfacing as the demand escalates for executive education that is adaptable, monitorable, and demonstrably effective. Various institutions, including business schools, consultancies, corporate universities, and digital platforms, are all contending to offer skills development programs. Each of these institutions has unique strengths and limitations within this competitive landscape. Continuous evaluation and feedback are key to fine-tuning the program and ensuring its alignment with the evolving digital transformation landscape (e.g., Suksai et al., 2021). A tool like the aforementioned quick-scan leadership assessment tool could support this.

### 5.3. Limitations and future research

The present study primarily concentrated on digital transformation as a distinct form of radical change, explicitly emphasizing the experiences of professionals within the largest public organization in the Netherlands. These professionals were undergoing digital transformation and participating in an education program centered on leadership and intelligence. Although the sample size was limited, the stress values remained within acceptable bounds (Rosas and Kane, 2012), suggesting the results possess both validity and reliability. Nonetheless, further data collection and refinement of the conceptual framework are warranted.

A noteworthy aspect of digital transformation, which sets it apart from other organizational transformations, is the speed and scale of the change and the fact that technology is the driver of the change (Berman, 2012; Westerman et al., 2014; Raz and Barnes, 2018). These unique characteristics present middle managers with a distinct set of challenges as they must navigate the complexities of digital innovations and their implications for organizational processes, culture, and strategy (e.g., Schallmo et al., 2017; Klein, 2020). Consequently, it is essential for future research to explore the specific ways in which digital transformation differs from other types of organizational change and how these differences influence middle managers’ leadership skills and behaviors.

To bolster the external validity and applicability of the findings, future investigations should incorporate cross-sectional data from organizations of diverse sizes and sectors. Such an approach would yield valuable insights into how sector-specific challenges and dynamics impact middle managers’ leadership skills and behaviors during digital transformations. Moreover, researchers should examine additional demographic factors and sources of heterogeneity, including management layers, personal values, and individual personality traits, to comprehensively understand the interplay between these variables and leadership behaviors amid digital transformations. Given the aging demographic of the workforce in the Netherlands, it becomes increasingly important to concentrate on how these mature employees can enhance their digital and soft skills (Oude Mulders et al., 2020).

Future research should also consider the temporal dimension of digital transformations by employing longitudinal study designs. This approach would facilitate examining the evolution and interaction of middle managers’ leadership conceptualizations over time, shedding light on the trajectories of leadership development and adaptation in response to ongoing digital transformations (Schwarzmüller et al., 2018).

Additionally, it is crucial for future studies to explore the influence of organizational culture, communication channels, and decision-making processes on the success of digital transformation initiatives, as these factors may significantly contribute to the overall efficacy of middle managers’ leadership endeavors.

Lastly, future studies should investigate the role of context in shaping leadership skills and behaviors during digital transformations. By examining how various environmental factors—such as technological infrastructure, regulatory frameworks, and socio-cultural norms—affect the nature and outcomes of digital transformation efforts, researchers can elucidate the intricate interplay between leadership and digital transformation. This, in turn, would provide valuable insights for organizations navigating the disruptive changes brought about by digital transformations.

### 5.4. Conclusion

By using the group concept mapping method we were able to gain insight into which leadership skills and behaviors middle managers in practice consider important during ongoing digitalization. The results revealed six clusters covering an array of leadership skills and behaviors, with soft people-oriented skills being considered the most important. In combination with a growing number of—predominantly theoretical—studies on this topic, this participant-driven study, deepens our understanding of middle managers’ leadership skills and behaviors in relation to digital transformation and supplements extant leadership research on radical change in general. It also highlights the need for middle managers to develop these skills for instance, via a leadership development program. An effective leadership development program should prioritize soft skills development, focusing on teamwork, collaboration, communication skills, and individual personalities but also include understanding the dynamic digital landscape.

## Data availability statement

The raw data supporting the conclusions of this article will be made available by the authors, without undue reservation.

## Author contributions

MH and JS contributed to conceptualization, investigation, methodology, formal analysis, validation, and writing, including the original draft’s writing, reviewing, and editing. All authors contributed to the article and approved the submitted version.

## Conflict of interest

The authors declare that the research was conducted in the absence of any commercial or financial relationships that could be construed as a potential conflict of interest.

## Publisher’s note

All claims expressed in this article are solely those of the authors and do not necessarily represent those of their affiliated organizations, or those of the publisher, the editors and the reviewers. Any product that may be evaluated in this article, or claim that may be made by its manufacturer, is not guaranteed or endorsed by the publisher.
