# Supplementary material for: Digital transformation and middle managers’ leadership skills and behavior: a group concept mapping approach
Source: Front Psychol. 2023 Sep 4;14:1147002. doi: 10.3389/fpsyg.2023.1147002 (PMC10507628; doi:10.3389/fpsyg.2023.1147002)
Supplement: Supplementary file 1 [file Table_1.pdf]

Appendix A. Overview of statements per cluster

Cluster 1 - Improvements and results	
1	Support online teams*
32	Lead by result[§]
41	Stimulate remote working
53	Taking opportunities to improve work
54	Evaluate digital work
55	Encourage efficient work
67	Able to structure workflows
72	Focus on performance[§]
74	Ensure work-life balance
77	Process monitoring[§]
Cluster 2 - Digital technology	
24	Aware of the possibilities of informatics and robotics
29	Thinking in terms of profitability
35	Knowledge of digital tools/assets/software/hardware
36	Recognizing the value of digital tools
37	Encouraging use of digitals tools and resources
51	Recognizing benefits of digital technology
64	Critical of the added value of digitalization
68	Aware of the influence of digitalization
75	Support employees to cope with responsibilities of the freedoms that digitalization offers
76	Monitoring online privacy and security
78	Able to structure information using digital possibilities
79	Able to structure processes with the help of digital possibilities
80	Support employees to accept digitalization
82	Being able to use digital tools to simplify processes
86	Seek connections between online and offline contexts
90	(Digital) information literate
92	Making the usefulness of digitalization visible
Cluster 3 - Cooperation	
38	Investing in external cooperation regarding surveillance in digital environments
39	Investing in external cooperation regarding enforcement in digital environments
40	Investing in public-private cooperation in learning and development
43	Stimulating internal cooperation[☺]
56	Encouraging staying informed about each other's work
57	Stimulating internal collaboration
58	Fostering interpersonal communication[☺]
91	Encouraging international cooperation
Cluster 4 - Self	
2	Critical thinking*
4	Open to feedback*
5	Authentic*
8	Integrity*
10	Vulnerable*
11	Humbleness*
12	Creativity*
13	Patient*
14	Tolerant*
15	Empathic*
16	Broad-minded*
17	Emotional intelligence*

20	Adaptability*
22	Open to innovation*
25	Restraint
27	Realistic
31	Trust
46	Convincing
48	Inspiring
60	Thoughtful
61	Sympathetic
62	Flexible
63	Open minded
66	Committed
70	Dedicated
85	Curiosity
87	Courageous
89	Good sense of ethics
94	Understanding
Cluster 5 - Change and ambivalence	
18	Teambuilding skills*
19	Dealing with change*
21	Unafraid to fail*
23	Open to experimenting*
26	Sharp focus
33	Adaptable leadership style
34	Consideration across generations
52	Recognizing opportunities
59	Making quick decisions
65	Offering custom solutions
84	Coping with resistance
88	Pursuing a higher goal
Cluster 6 - Others	
3	Communicating effectively*
6	Relationship orientation*
7	Coping with uncertainty*
9	Sharing responsibilities*
28	Encouraging out-of-the-box thinking
30	Facilitating learning and development
42	Maintaining personal contact
44	Enthusing employees
45	Motivating
47	Stimulating
49	Attention on the individual
50	Thinking in possibilities
69	Empowering people
71	Creating connections between people
73	Networking skills
81	Showing sustainable behaviors
83	Sensitive to feelings
93	Supporting employees with coping with change

Note. *Statements from theory; [§]Statements reassigned from Cluster 5; [☺]Statements reassigned from Cluster 6.
